# Zygomatico-Coronoid Pseudoarthrosis Due to Osteochandroma of Coronoid Process: A Rare Entity

**DOI:** 10.22038/ijorl.2021.48888.2617

**Published:** 2021-05

**Authors:** Mehtab Alam, Syed Abrar Hasan, Kamlesh Chandra

**Affiliations:** 1 *Department* of Otorhinolaryngology, Faculty of Medicine, Aligarh Muslim University, Aligarh, India.

**Keywords:** Jacob’s disease, Osteochondroma, Temporo-mandibular disorders, 3D computed tomography, Coronoidectomy

## Abstract

**Introduction:**

Osteochondroma of the coronoid process also known as Jacob’s disease, has rarely been reported in the literature and it posses a challenge as diagnosis may be overlooked in favour of other causes of limited mouth opening.

**Case Report:**

This is a case report of an adult male patient who presented with restricted mouth opening in whom radiological imaging, especially 3D computed tomography, played a role in establishing the diagnosis of Jacob’s disease.

**Conclusion:**

An osteochondroma of the coronoid process of the mandible (Jacob’s disease) is a rare cause of restricted mouth opening and its diagnosis can be overlooked in favour of TMJ ankylosis. The CT scan plays an important role in diagnosis and in planning for surgery.

## Introduction

Osteochondroma, or osteocartilaginous exostosis as it may be called, is a cartilage-capped mass arising from the cortex of a bone and constitutes around 10–15% of all bone tumours (1). It is more frequently found in long bones due to endochondral growth and may involve the facial bones, with involvement of the coronoid process of the mandible being rare. The enlargement of the coronoid process of the mandible was first described by Langenbeck in 1853 and may be caused by osteochondroma, exostosis, osteoma, hypertrophy and developmental anomalies (2). Jacob was the first to describe joint formation between the coronoid process and zygoma (3, 4). Until 2018 only around 38 histologically proven cases of osteochondroma of the coronoid process of the mandible had been documented in the literature (5). The disease shows sex predilection with the male population (73.5%) being affected more than the female population, with a mean age of around 35 years (6). Surgery is the only modality of treatment, with the intraoral approach being the preferred one without any reported recurrence.

## Case Report 

A 32-year-old man presented to our outpatient department with complaints of progressive restricted mouth opening and painless swelling in the right zygomatic region since the past 6 months. On physical examination there was facial asymmetry with protruding right zygoma. The swelling was diffuse with osseous consistency and non-tender with normal overlying skin. The inter-incisal opening was only 8 mm (Figure 1a). Radiography of the bilateral temporomandibular joint (TMJ) was normal without any signs of ankylosis.

**Fig 1a:**
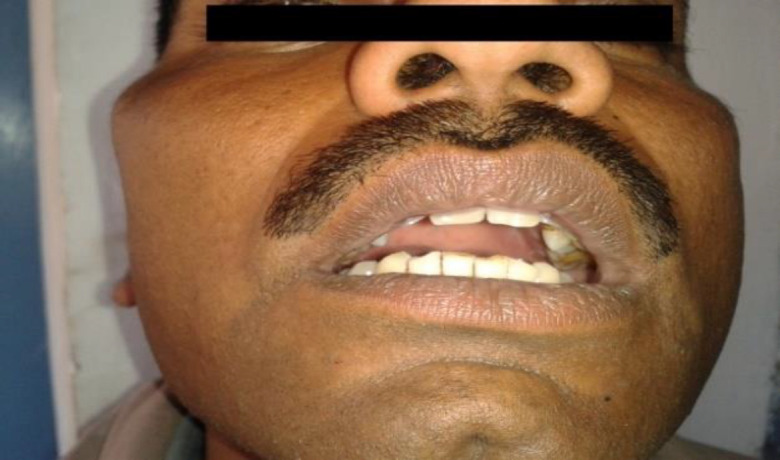
Preoperative picture showing facial assymetry and decreased inter-incisal jaw opening

Computed tomography imaging of the face revealed an elongated coronoid process of the mandible forming a pseudojoint with the inner surface of the zygoma; there was also thinning of the zygomatic arch (Fig. 2a and 2b). Hence, radiological diagnosis of Jacob’s disease was made pre-operatively. The patient was counselled for surgery and an external approach using modified hockey stick incision was adopted. Because of restricted mouth opening nasotracheal intubation was performed using a fibreoptic laryngoscope. 

A fibrous pseudocapsule was found around the mass and was released. Coronoidectomy was performed with around 1 cm of healthy bone below the tumour margins. Grossly, the resected mass measured 2.5 cm and consisted of bone covered by a cap of cartilage (Figure 3). Histopathological examination revealed findings consistent with osteochondroma, which comprises irregularly arranged fibrous, bony and cartilaginous elements (Fig. 4).

On the seventh postoperative day the measured inter- incisal opening was 38 mm (Figure 1b), and swelling over the zygomatic area was minimal. The patient was instructed to perform mouth opening exercises using spatulas. 

**Fig 1b:**
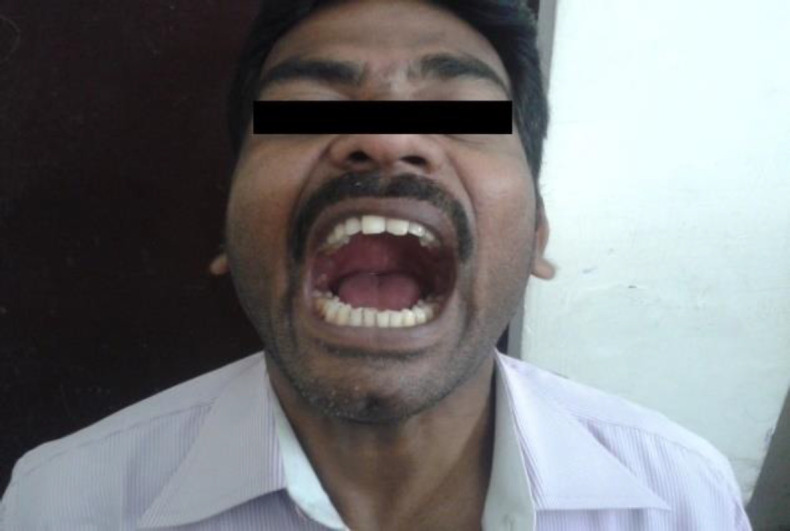
Seventh postoperative day picture with increased jaw opening and absent asymmetry

**Fig 2a:**
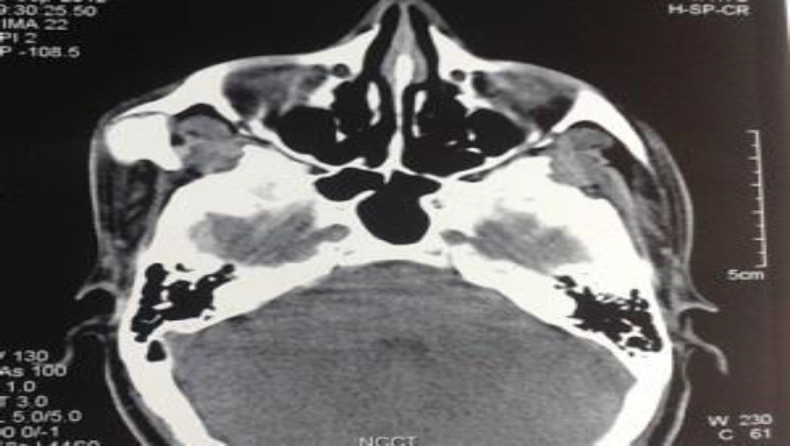
Axial Computed tomographic image revealing elongated coronoid causing thinning and bulging of zygoma

**Fig 2b:**
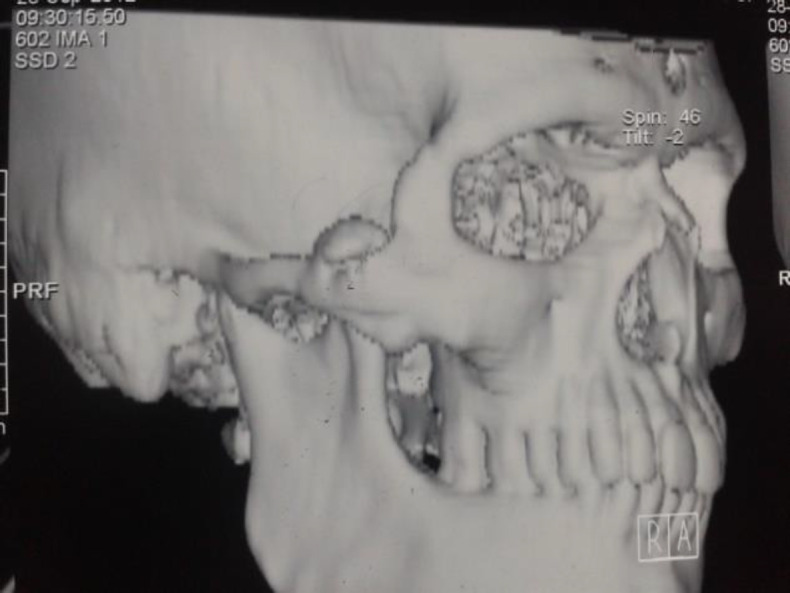
Computed tomography with 3D reconstruction, hyperplastic coronoid posterior to zygomatic arch with pseudojoint formation

**Fig 3:**
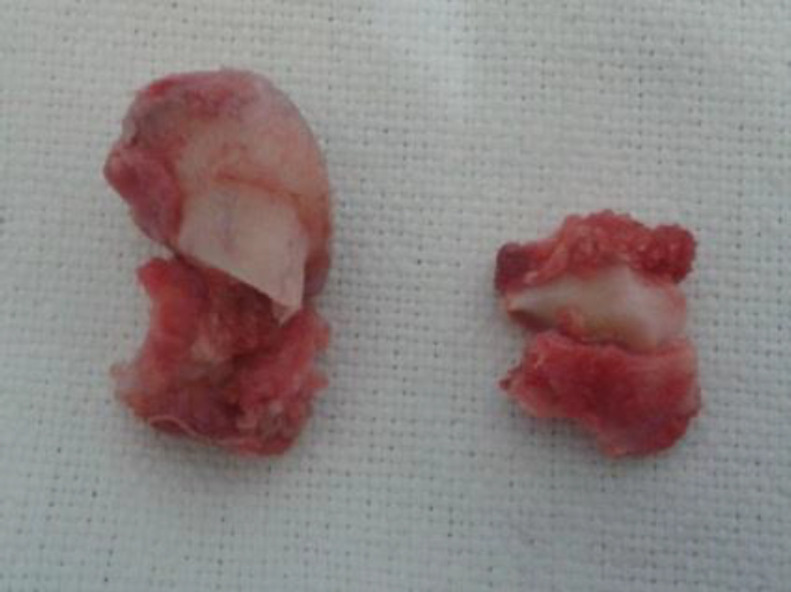
Surgical specimen showing cartilaginous cap over bone

**Fig 4:**
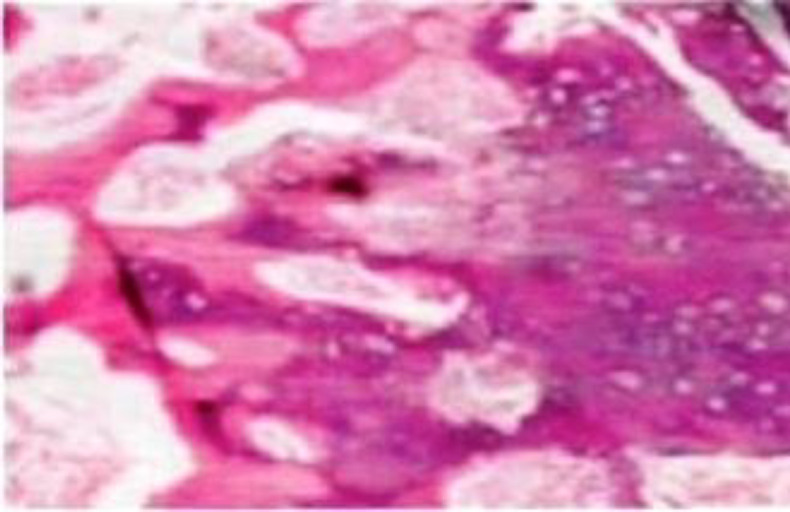
Histopathological picture showing irregularly arranged fibrous, bony and cartilaginous elements

## Discussion

Osteochondromas being the most common benign tumours of bones are relatively uncommon in the jaw, affecting the condyle or tip of the coronoid process. In the literature only 38 cases of histologically proven osteochondroma of the coronoid process have been documented (5), and also the same number of cases of this benign tumour had been reported affecting the mandibular condyle (7,8). In some cases this osteochondroma may primarily arise from the zygomatic arch and interfere with the coronoid process (9). Rarely this may undergo sarcomatous degeneration (10). This cartilage-capped growth can be an incidental finding on radiological examination or on palpation of a protruding mass in the affected area. The affeced persons initially present with gradual reduction in mouth opening, and in later stage there is painless facial mass with some trismus (6).

Although the exact causative factors are not known, several theories have been proposed to explain its aetiology. According to Weinmann and Sicher there is hyperplastic development of embryonic cells with chondrogenic potential because of continuous activity of tendons inserted in the coronoids (11). Litchtenstein proposes that the periosteum has pluritpotential to produce cartilage and osseous tissue (12). Other causes could be trauma and functional alterations in the shape and structure of the coronoid process.

Most of the patients with coronoid process hyperplasia are first diagnosed as having a TMJ disorder and managed as such. Magnetic Resonance Imaging of the TMJ does not evaluate the coronoid processes because they are not included in the field of view (13). Although Water’s radiograph is useful for showing enlargement of the coronoid process and its relation to the zygoma, for establishing the diagnosis of Jacob’s disease there should be a direct contact between the hyperplastic coronoid and the posterior wall of the maxilla or zygomatic arch and joint formation at this site. This is best seen with 3D reconstructed computed tomography (3), as in this case (Figure 2b ).

The primary goal of treatment is to recover acceptable mouth opening. The surgical approaches described can either be intraoral, external, or a combination of both (2). Until 1961 almost all of the reported cases were resected through the external zygomatico-facial approach with or without separation of the zygomatic arch. However, there is risk for facial nerve injury and sometimes unacceptable facial scar. The intraoral approach first described by Antoni (1958) is commonly used and is safe and allows complete removal of the tumour (14).

In this case the external approach using modified hockey stick incision was adopted as the patient presented with markedly restricted mouth opening, there was thinning of the zygoma, and the mass was felt superficially. Post- operatively, the patient had near-normal mouth opening and acceptable facial scar without any other complication.

## Conclusion

An osteochondroma of the coronoid process of the mandible (Jacob’s disease) is a rare cause of restricted mouth opening and its diagnosis can be overlooked in favour of TMJ ankylosis. The CT scan plays an important role in diagnosis and in planning for surgery. The intraoral approach is most favoured because of minimal or no complications, but in some cases the size and bulbous nature of the tumour may prevent delivery of the mass through the space between the zygomatic arch and the temporal bone, and in such cases an external approach is indicated.
